# Biological Effects of Anodic Oxidation on Titanium Miniscrews: An In Vitro Study on Human Cells

**DOI:** 10.3390/dj7040107

**Published:** 2019-11-17

**Authors:** Giorgio Iodice, Giuseppe Perinetti, Bjorn Ludwig, Elena V. Polishchuk, Roman S. Polishchuk

**Affiliations:** 1 Private Practice, 81100 Caserta, Italy; iodicegiorgio@gmail.com; 2 Private Practice, 65010 Nocciano (PE), Italy; 3 Private Practice, 56841 Traben-Trarbach, Germany; bludwig@kieferorthopaedie-mosel.de; 4 Telethon Institute of Genetics and Medicine, 80078 Pozzuoli (NA), Italy; epolish@tigem.it (E.V.P.); polish@tigem.it (R.S.P.)

**Keywords:** titanium, anodic oxidation, miniscrews, orthodontics, cell growth

## Abstract

This controlled in vitro study compared the effects of varying the thickness of a TiO_2_ layer on cellular activity using commercially available miniscrew samples with identical surface features to derive information with direct clinical impact. Titanium grade V plates with four different thicknesses of TiO_2_ layer/color were used: absent/gray (Control group), 40–50 nm/pink (Pink group), 130 nm/gold (Gold group) and 140 nm/rosé (Rosé group). In vitro experiments used Saos-2 cells and included cell growth analysis, phospho-Histone H3 and procollagen I staining, cell viability analysis, and a cell migration assay at 12, 24, 40 and to 48 h. Few differences were seen among the groups, with no clear behavior of cellular activity according to the TiO_2_ thickness. The Control group showed a greater cell count. Phospho-Histone H3 staining was similar among the groups and procollagen I staining was greater in the Rosé group. Cell viability analysis showed a significant difference for live cell counts (greater in the Rosé group) and no difference for the dead cell counts. The cell migration assay showed a delay for the Rosé group up to 40 h, where full repopulation of cell-free areas was obtained at 48 h. The results suggest that the TiO_2_ layers of the commercial miniscrews have minimal biological effects, including cytotoxicity, with possibly negligible or minimal clinical implications.

## 1. Introduction

Cellular response to titanium implants or miniscrews depends on the topography, chemical composition and other features of the implant surface, which in turn have an impact on adhesion, proliferation, migration, survival, and differentiation (for review, see Ref. [1]). While it is known that titanium is an ideal material for orthopedic and dental implants, a significant amount of research has been focused on augmenting the therapeutic efficacy of titanium surfaces [2,3].

Among these procedures is the recently introduced anodic oxidation, which is obtained by immersion of the titanium in an electrolyte solution and by the application of high current voltage [4,5]. Upon anodic oxidation, the titanium surface is covered by a TiO_2_ layer [4,5], organized in self-ordered nanotubular structures [2] vertically oriented on the titanium surface with a closed bottom and open top [6]. Notably, the greater the applied current voltage, the thicker and more rough the TiO_2_ layer will be [7]. This TiO_2_ layer increases surface roughness, so that anodic oxidation has been reported to yield titanium surfaces with similar roughness behavior to that of machine treated implants [8]. 

Variable thickness of the TiO_2_ layer is also responsible for different surface coloration, which is an effect of light refraction [9]. Therefore, anodic oxidation is becoming a common procedure in manufacturing orthodontic miniscrews that carry a specific color according to their lengths. However, in spite of the commercial availability of miniscrews treated by anodic oxidation and their wide use in orthodontics [10], evidence on the biological effects of such a TiO_2_ layer for orthodontic purposes is still scarce. Although the cellular response to TiO_2_ has been investigated using a variety of cell types, including osteoblasts, fibroblasts, and chondrocytes (for review, see Ref. [11]), no previous studies have evaluated the behavior of commercially available products, including the effects of varying the thickness of the TiO_2_ layer. Specifically, previous studies on orthodontic miniscrews are limited to the analyses of implant surfaces [5], insertion/removal torque and bone-to-implant contact sites [6,7,8]. 

Therefore, this controlled in vitro study compared the effects of different thickness of TiO_2_ on cellular activity using commercially available miniscrew samples with identical surface features. The null hypothesis was that the thickness of TiO_2_ had no impact on cellular activity (as recorded through different parameters). Derived implications regarding positive or negative biological effects are thus expected to be of direct relevance both for future research and clinical activity.

## 2. Materials and Methods 

### 2.1. Titanium Plates, Cell Culture and Reagents

**Figure 1:**
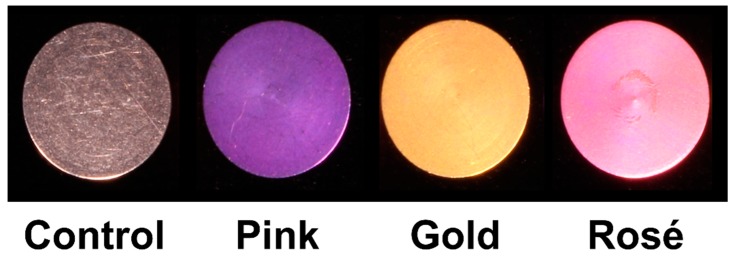
The different plates used in the study.

For the present study, circular 10-mm-diameter (2-mm thick) titanium grade V plates were used after being rinsed with distilled water and sterilized overnight by UV irradiation. No further manipulation was executed. The plates were classified according to the thickness of their TiO_2_ layer/color: absent/gray (Control group), 40–50 nm/pink (Pink group), 130 nm/gold (Gold group) and 140 nm/rosé (Rosé group) (Figure 1). These plates had identical properties in terms of titanium and TiO_2_ layer with respect to commercially available orthodontic miniscrews (Orthoeasy^®^, pink [1101A2308], gold [1101A2310] and rosé [1101A2306], Forestadent GmbH, Pforzheim, Germany). A total of 272 plates (68 per type) were employed.

Human osteoblast-like osteosarcoma Saos-2 cells were grown in McCoys medium supplemented with 15% fetal bovine serum, 2 mM L-glutamine, 1% penicillin and streptomycin. The following antibodies were used: (i) rabbit anti-phospho-Histone H3 (pSer10, D2C8 XP^®^ Rabbit mAb #3377, Cell Signaling Technology, Leiden, The Netherlands) as a marker of cells undergoing mitosis; (ii) mouse anti-procollagen I (SP1.D8, Antibody Registry ID: AB_528438, Developmental Studies Hybridoma Bank, Iowa City, IA) as a marker of collagen synthesis; (iii) mouse anti-α-tubulin (T5168, Sigma-Aldrich^®^, St. Louis, MO, USA) to visualize microtubules, thus helping detection of the entire shape of the cells; and (iv) secondary Alexa Fluor 488 goat anti-rabbit (A-11034) and goat anti-mouse (A-11029) and secondary Alexa Fluor 568 goat anti-rabbit (A-11011) and goat anti- mouse (A-11031) IgG antibodies (Life Technologies, Thermo Fisher Scientific, Waltham, MA, USA). Finally, Hoechst 33342 Solution (62249, Thermo Fisher Scientific) was used to stain the nuclei (along with the used secondary antibody). All the reagents were used according to the manufacturers’ instructions.

### 2.2. Fixing, Blocking and Staining with Primary and Secondary Antibodies

In all the samples, cells were fixed with 4% paraformaldehyde in 0.15 M HEPES buffer, pH 7.4, for 10 min at room temperature, and then washed six times with phosphate-buffered saline (PBS). Cells were then permeabilized with blocking solution containing 50 mM NH4Cl, 0.1% saponin, 0.5% bovine serum albumin (BSA) in PBS, pH 7.4, for 30 min at room temperature. Subsequently, the samples were incubated with the specific primary antibody (according to manufacturer’s instruction, see above) in blocking solution for at least 1 h at room temperature. After incubation with the primary antibody, the cells were washed six times with PBS, to remove excess primary antibody, and then incubated with the secondary antibody (according to manufacturer’s instruction, see above), again in blocking solution, for 1 h at room temperature and protected from light. The excess secondary antibody was removed by washing the samples with PBS. Each sample was then put into a 35-mm petri dish upside down (with cells at the bottom) to allow visualization under microscopy through the transparent side (bottom of the petri dish).

### 2.3. Cell Growth Analysis, Phospho-Histone H3 and Procollagen I Staining

A total of 150,000 cells were plated on each of the 10 different plates per group and grown for 24 h, after which the different analyses were carried out. For cell growth analysis, cells were fixed with 4% paraformaldehyde and labeled with a common blue fluorescent DNA dye Hoechst to recognize the nuclei. Images were acquired by an LSM 700 confocal microscope (Carl Zeiss Microscopy GmbH, Göttingen, Germany). For each experiment, the number of cells was recorded in duplicate (1 week apart) in equal fields of view (408,218.76 μm^2^) on each sample with the mean value representing the statistical unit. The number of cells was calculated by counting the cell nuclei stained with Hoechst using the program ImageJ (version 1.8.0, https://imagej.nih.gov/ij/). For the phospho-Histone H3 and procollagen I staining (performed according to the manufacturers’ instructions for each antibody used), mean fluorescence intensity in arbitrary units (au) was recorded.

### 2.4. Cell Viability Analysis

To analyze cell viability, the Live/Dead™ Viability/Cytotoxicity Kit (L3224, Invitrogen™, Thermo Fisher) was used. A total of 150,000 cells were plated on each of the 8 different plates per group and grown for 24 h, after which they were incubated with the reagent for 30 min at room temperature, washed in Dulbecco’s PBS and immediately analyzed using the LSM 700 confocal microscope. The reagent allows for the distinction of live (producing green fluorescence) and dead (producing red fluorescence) cells. The number of both live and dead cells was recorded in duplicate (1 week apart) in equal fields of view (408,218.76 μm^2^) on each sample with the mean value representing the statistical unit. The number of dead cells as a percentage of total cells was also calculated.

### 2.5. Cell Migration Assay

For the cell migration assay, a culture-Insert-2-Well (81176, ibidi GmbH, Martinsried, Germany) was used in combination with the different plates. Two-hundred-thousand cells were plated in each well separated from each other by a 500-µm-wide-silicon-insert gap and left for growing. The following day, the 2-well silicon insert was removed to allow cell migration towards the defined 500-µm-wide-cell-free area for a further 12 h, 24 h, 40 h and 48 h time course. At each time course in 10 plates per group, cells were fixed, labeled with Hoechst and α-tubulin antibody and analyzed under the confocal microscope. The areas (in µm^2^) that remained free from migrating cells were recorded using the ZEN 2008 software (Carl Zeiss Microscopy GmbH). The total surface of the investigated area per sample (including cell-free and cell-covered ones) was defined as a square of side 500 µm, corresponding to 250,000 µm^2^. Areas were recorded in duplicate (1 week apart) on each sample, with the mean value representing the statistical unit. An attempt was made to exclude from this recording areas for which the gap was originally larger than 500 µm.

### 2.6. Data Analysis

The Statistical Package for Social Sciences Software 20.0 (SPSS Inc., Chicago, IL, USA) was used for data analysis. Each data set was tested for the normality of the data by means of the Shapiro–Wilk test and by Q-Q normality plots. Equality of variance was also tested by means of the Levene test and Q-Q normality plots of the residuals. Each dataset was treated as ordinal data by non-parametric tests, due to the failure to meet the required assumption for using parametric analyses (as revealed though the Shapiro–Wilk and Levene tests). Nevertheless, the mean and standard deviations are reported for descriptive purposes, along with the median and range for most of the data sets. For each parameter, the significance of the differences across groups (and the different time course recordings) was assessed using a Kruskal–Wallis test followed by a Bonferroni-corrected Mann–Whitney U-test, where appropriate [12]. A *p* value less than 0.05 was used for rejection of the null hypothesis. Raw data file is available as a supplementary file.

## 3. Results

Results from the cell growth analysis and phospho-Histone H3 and procollagen I staining are summarized in Table 1. The total cell count (n) ranged from 156.1 ± 18.4 to 250.5 ± 22.3 in the Pink and Control groups, respectively. The difference among the groups was statistically significant (*p* = 0.000), with the Control group showing a significantly greater cell count compared to the other groups. Phospho-Histone H3 staining (in au) ranged from 1.6 ± 1.1 to 1.9 ± 1.3 in the Pink and Control groups, respectively. No significant differences among the groups were seen. Procollagen I staining (in au) ranged from 19.3 ± 5.0 to 30.9 ± 10.4 in the Pink and Rosé groups, respectively. The difference among the groups was statistically significant (*p* = 0.019), while no significant differences were retrieved in the pairwise comparisons.

Results of the cell viability analysis are summarized in Table 2. Live cell count (*n*) ranged from 55.3 ± 24.4 to 82.1 ± 18.2 in the Gold and Rosé groups, respectively. The difference among the groups was statistically significant (*p* = 0.016), while no significant differences were retrieved for the pairwise comparisons. Dead cell count (*n*) ranged from 8.6 ± 6.5 to 25.6 ± 27.3 for the Control and Rosé groups, respectively. The difference among the groups was not statistically significant (*p* = 0.062). The number of dead cells as a percentage of the total cells present (%) ranged from 11.3 ± 8.2 to 23.4 ± 14.2 in the Control and Gold groups, respectively. The difference among the group was not statistically significant (*p* = 0.086).

**Figure 2:**
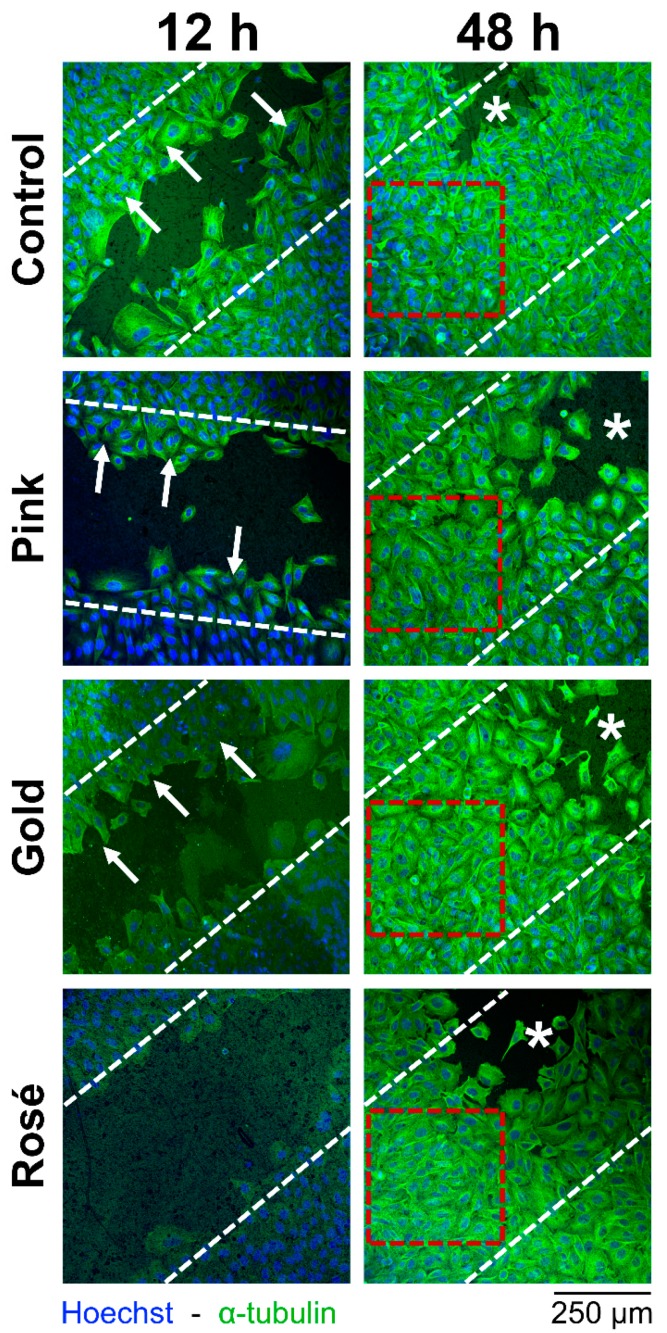
Representative cell migration assay images at 12 and 48 h, showing the different groups. Smaller cell-free areas in images indicates better migration of cells. White dashed lines indicate the 500-µm cell-free area which existed after the removal of the silicon insert. Arrows identify Saos-2 migrating cells on the cell-free area during the initial repopulation of the cell-free area at 12 h. Red dashed squares designate full repopulation of the cell-free area at 48 h. Asterisks indicate areas for which the gap was originally larger than 500 µm (excluded from the recordings). Images at baseline, 24 h and 40 h omitted for clarity.

Results of the cell migration assay are summarized in Table 3, with representative images shown in Figure 2. At 12 h, the cell-free area (in µm^2^) ranged from 97.5 ± 24.1 to 212.7 ± 24.3 in the Control and Gold groups, respectively. The difference among the groups was statistically significant (*p* = 0.000), with the Control group showing a significantly lower value compared to the other groups. At 24 h, the cell-free area ranged from 28.0 ± 25.7 to 122.6 ± 73.1 in the Control and Rosé groups, respectively. The difference among the groups was statistically significant (*p* = 0.019), with the Rosé group showing a significantly greater value compared to the Control group. At 40 h, the cell-free area ranged from 0.3 ± 0.5 to 28.8 ± 22.2 in the Control and Rosé groups, respectively. The difference among the groups was statistically significant (*p* = 0.005), with the Rosé group again showing a significantly greater value compared to the Control group. At 48 h, no cell-free area was detected in any group.

## 4. Discussion

Anodic oxidation treatment may be an effective tool in reducing insertion damage to surrounding tissue and improving the mechanical stability of miniscrews [4] by increasing bone-implant contact [8]. Previous studies analyzing the response of cells to TiO_2_ made use of different specific anodization processes [6,13,14], processes which yield very different surface treatments that are likely not comparable to those investigated here. Therefore, the biological effects of the thickness of the TiO_2_ layer in commercial miniscrews is still to be analyzed [8].

When inserted, orthodontic miniscrews are mostly in contact with bone tissue (with only a minor part at the collar in contact with gingival tissue). The stability of the miniscrews thus depends critically on bone integrity. This is the reason why human osteoblast-like cells were chosen for the present investigation, instead of epithelial or other cells.

While total cell count acts as an overall index of cell activity, type I collagen, derived from procollagen I, is one of the major structural components of the bone extracellular matrix, and may therefore be used as an index of the structural and adhesive functions of the cells [15]. Total cell count and procollagen I staining were significantly different among the groups. However, only for the former were significant differences seen in the pairwise comparisons, with the Control group having the greatest number of cells. For both parameters, the lowest scores were retrieved from the Pink group, followed by the Gold group (Table 1). Even though absence of TiO_2_ favored the cell count, there was no clear behavior in these parameters according to the different thicknesses of the TiO_2_. Phospho-Histone 3 was used in this study as a marker of mitotic activity [16]. Contrary to other parameters, no significant difference was seen in its staining, with very similar values obtained among the groups, indicating that mitosis was not influenced by the presence of the different TiO_2_ layers.

In the cell viability analysis (Table 2), only the live cell count showed a significant difference among the groups, while the dead cell count and number of dead cells as a percentage of the total cells present were similar among the groups (although close to the statistical significance level). Even though the Rosé group showed more live cells, this group also showed more dead cells and a greater percentage of dead cells (close to the statistical significance level). Of note, the sum of live and dead cell counts for the Pink and Rosé groups was greater than those seen for the other groups (Table 2). This apparent inconsistency is due to the fact that only attached cells (live or dead) were included in the count. It might be possible that the different surfaces had an impact on the number of attached cells. Moreover, a certain variability among experiments and sample is expected and this can also, at least partially, explain apparent inconsistencies. Therefore, clear behavior of the different groups according to the thickness of the TiO_2_ layer was not seen. Notably, the Rosé group showed the greatest variability (i.e., standard deviation) in the dead cell parameters, decreasing the reliability of the slight differences observed.

Inconsistencies with previous in vitro studies, which report better cellular activity [11,14] and protein secretion [3] on a TiO_2_ surface, may be explained by the noteworthy differences in carrying out the process of anodic oxidation. For instance, other than different current voltages [13] or durations of application [3], the modality through which it is provided—such as constant [17] or pulse [7]—and the duration of the anodizing process [7,13,17] have relevant effects on the final thickness of the TiO_2_ layer and nanoscale surface roughness. The composition of the electrolyte solution also affects the surface composition, specifically its retainment of calcium and phosphorus [7]. Moreover, the thicknesses reported in previous investigations were notably greater than those investigated here, being 300 nm [14], ~3 µm [7] and ~5 µm [6]. This may explain why the samples investigated in this study, which had thinner TiO_2_ layers (between 40 and 140 nm), had behavior similar to that of the Control group for several, although not all, parameters.

For the cell migration assay (Table 3), the Control group and Rosé group generally showed more rapid migration and slower migration, respectively. Significant differences were seen among the groups at 12, 24 and 40 h, but no cell-free areas were seen in any of the groups at 48 h of repopulation. In contrast, accelerated cell adhesion and growth on TiO_2_, as compared to a titanium surface, has been reported for MC3T3-E1 mouse preosteoblasts [14,18]. This inconsistency may be explained by the different cell lines or the surface roughness. Indeed, previous studies have shown that microscale surface roughness of 1–5 µm augments bone-to-implant contact [19] and the removal torque of the dental implant [20]. On the contrary, miniscrews that have undergone anodic oxidation resulting in nanoscale surface roughness (~133 nm) fail to show greater removal torques or bone-implant contact values [8]. Nevertheless, initial biomolecular interactions and cell behavior can be enhanced or delayed by a nanoscale surface roughness <100 nm or >100 nm, respectively [3,13]. This evidence may explain the slightly different results seen for the Rosé group (with the thickest TiO_2_ layer) in terms of the cell migration assay, where full repopulation of the cell-free area was achieved later than the other groups. (Table 3, Figure 2). However, whether this delay in migration has a clinical implication has yet to be verified.

The present study, however, suffers limitations, as a full analysis of the surface features of the TiO_2_ layers is missing. Such analyses would include, for instance, surface roughness, ultrastructural cell analysis or even scanning electron microscopy of the cell adhesion. Even though inclusion of these analyses would not change the conclusions of the present investigation, future studies are warranted to fully elucidate what sort of interactions exist between the different TiO_2_ layers and the populating cells.

While the main advantage of anodic oxidation is to give a specific color to each of the different products for easy recognition, the present study suggests that the TiO_2_ layers of the commercial miniscrews investigated here would have minimal biological effects and possibly negligible or minimal clinical implications, as no clear cellular behavior in response to the different groups was recorded.

## 5. Conclusions

- Although some minor effects of the TiO_2_ were detected on cell viability, these may be limited to the very early stage of surface population.
- No clear relationship between the thickness of the TiO_2_ layers and the degree of cellular response was recorded.

## Figures and Tables

**Table 1:** Cell growth analysis (as total cell count) and phospho-Histone H3 and procollagen I staining (as mean fluorescence) results for the different groups.

Parameter, Group	Mean ± SD	Median	Min-Max
Total cell count (*n*)			
Control	250.5 ± 22.3 (a)	254.0	208.5–277.0
Pink	156.1 ± 18.4	160.3	120.5–180.0
Gold	163.0 ± 24.8	152.8	138.5–212.5
Rosé	213.0 ± 23.1	216.8	175.0–240.5
Diff.	0.000		
Phospho-Histone H3 (au)			
Control	1.9 ± 1.3	1.5	0–3.5
Pink	1.6 ± 1.1	1.5	0–4.0
Gold	1.7 ± 0.9	1.8	0–2.5
Rosé	1.8 ± 1.2	1.8	0–3.5
Diff.	0.964		
Procollagen I (au)			
Control	27.1 ± 6.8	26.4	17.3–37.3
Pink	19.3 ± 5.0	19.3	11.9–26.5
Gold	24.9 ± 5.9	24.7	14.6–32.6
Rosé	30.9 ± 10.4	27.4	18.0–50.2
Diff.	0.019		

Statistical unit was mean of duplicate recordings (*n* = 10 each). *n*, number; au, arbitrary units. Diff., significance of the difference among the groups. a, pairwise comparisons the total cell count: significant difference of the Control group with Pink (*p* = 0.000), Gold (*p* = 0.000) and Rosé (*p* = 0.015) groups. Pairwise comparisons for the procollagen I staining were not significant.

**Table 2:** Cell viability analysis (as number of live and dead cells, and percentage dead cells on total) results for the different groups.

Parameter, Group	Mean ± SD	Median	Min-Max
Live cell count (*n*)			
Control	67.2 ± 10.2	66.5	51.5–80.5
Pink	79.4 ± 31.8	89.8	16.5–123.0
Gold	55.3 ± 24.4	57.3	21.5–85.1
Rosé	82.1 ± 18.2	88.8	43.5–97.5
Diff.	0.016		
Dead cell count (*n*)			
Control	8.6 ± 6.5	6.0	4.5–26.0
Pink	8.7 ± 6.4	9.0	1.5–23.5
Gold	19.1 ± 17.9	7.0	4.5–52.1
Rosé	25.6 ± 27.3	14.5	4.5–79.5
Diff.	0.062		
Death cells / total (%)			
Control	11.3 ± 8.2	8.9	5.6–33.5
Pink	12.6 ± 11.7	9.0	1.2–37.7
Gold	23.4 ± 14.2	22.7	6.3–47.8
Rosé	20.7 ± 17.0	13.6	6.5–58.5
Diff.	0.086		

Statistical unit was mean of duplicate recordings (*n* = 8, each) for every parameter. *n*, number. Diff., significance of the difference among the groups. Pairwise comparisons for the live cell count were not significant.

**Table 3:** Cell migration assay (as cell-free areas in µm^2^xE3) results for the different groups.

Group	Time Course			
	12 h	24 h	40 h	48 h
Control	97.5 ± 24.1	28.0 ± 25.7	0.3 ± 0.5	0
Pink	155.2 ± 30.5 (a)	51.4 ± 37.5	15.2 ± 22.4	0
Gold	212.7 ± 24.3 (a)	44.3 ± 21.4	3.7 ± 7.1	0
Rosé	160.9 ± 44.9 (a)	122.6 ± 73.1 (a)	28.8 ± 22.2 (a)	0
Diff.	0.000	0.019	0.005	--

Statistical unit was mean of duplicate recordings (*n* = 10, each) for every time point. Diff., significance of the difference among the groups within each time course. a, pairwise comparisons with significant difference of the Control group with Pink (*p* = 0.015), Gold (*p* = 0.0.03) and Rosé (*p* = 0.027) groups at 12 h, and with Rosé group at 24 h (*p* = 0.024) and 40 h (*p* = 0.0.03).

## Supplementary Materials

The following are available online at https://www.mdpi.com/2304-6767/7/4/107/s1, The SPSS file of the raw data.
